# Integrative relational machine-learning for understanding drug side-effect profiles

**DOI:** 10.1186/1471-2105-14-207

**Published:** 2013-06-26

**Authors:** Emmanuel Bresso, Renaud Grisoni, Gino Marchetti, Arnaud Sinan Karaboga, Souchet, Marie-Dominique Devignes, Malika Smaïl-Tabbone

**Affiliations:** 1 Université de Lorraine, LORIA, UMR 7503, Vandoeuvre-lès-Nancy, 54506, France; 2 INRIA, Villers-lès-Nancy, 54600, France; 3 Harmonic Pharma, Espace Transfert INRIA NGE, Villers-lès-Nancy, 54600, France; 4 CNRS, LORIA, UMR 7503, Vandoeuvre-lès-Nancy, 54506, France

**Keywords:** Relational machine learning, Data integration, Drug discovery, Data mining, Drug side-effects

## Abstract

**Background:**

Drug side effects represent a common reason for stopping drug development during clinical trials. Improving our ability to understand drug side effects is necessary to reduce attrition rates during drug development as well as the risk of discovering novel side effects in available drugs. Today, most investigations deal with isolated side effects and overlook possible redundancy and their frequent co-occurrence.

**Results:**

In this work, drug annotations are collected from SIDER and DrugBank databases. Terms describing individual side effects reported in SIDER are clustered with a semantic similarity measure into term clusters (TCs). Maximal frequent itemsets are extracted from the resulting drug x TC binary table, leading to the identification of what we call side-effect profiles (SEPs). A SEP is defined as the longest combination of TCs which are shared by a significant number of drugs. Frequent SEPs are explored on the basis of integrated drug and target descriptors using two machine learning methods: decision-trees and inductive-logic programming. Although both methods yield explicit models, inductive-logic programming method performs relational learning and is able to exploit not only drug properties but also background knowledge. Learning efficiency is evaluated by cross-validation and direct testing with new molecules. Comparison of the two machine-learning methods shows that the inductive-logic-programming method displays a greater sensitivity than decision trees and successfully exploit background knowledge such as functional annotations and pathways of drug targets, thereby producing rich and expressive rules. All models and theories are available on a dedicated web site.

**Conclusions:**

Side effect profiles covering significant number of drugs have been extracted from a drug ×side-effect association table. Integration of background knowledge concerning both chemical and biological spaces has been combined with a relational learning method for discovering rules which explicitly characterize drug-SEP associations. These rules are successfully used for predicting SEPs associated with new drugs.

## Background

Side effects are unwanted responses to drug treatment. Some side effects are adverse, while others are more tolerable. Many side effects are detected during clinical trials, and adverse side effects are often responsible for the high attrition rate of drug candidates. For example in 2008, the French Department of Industry estimated that only 1 drug out of 250 was approved by the FDA [1]. Beside toxicity, it is not desirable to prescribe for a long period drugs having side effects like nausea or headache. Moreover, not all side effects are detected during clinical trials. For example, the cardiotoxicity of benfluorex was only recently highlighted [2] even though benfluorex was approved in the 1970’s. Thus, early recognition of side effects is an important issue for drug development and safety.

To support side effect exploration, two main resources reporting their association with drugs have been developed. The FDA Adverse Event Reporting System (FAERS) stores the observed side effects reported directly by health care professionals and consumers. The SIDER database stores side-effect information mentioned on drug package inserts [3].

Two groups of studies have been conducted on side effects. On the one hand, side-effect information has been exploited for drug repositioning. For example, Campillos et al. [4] used a corpus-based side-effect similarity approach to show that pairs of drugs sharing similar side effects can have common targets. Thus, they use side-effect similarity to predict new targets for a drug. In a similar spirit, Takarabe et al. [5] used FAERS to define pharmacological drug-drug similarity and to predict unknown drug-target interactions from the integration of the pharmacological similarity and genomic sequence similarity of target proteins. At the disease level, Yang and Agarwal [6] proposed an approach based on the hypothesis that drugs sharing side effects could be indicated for the same disease. Drug side-effect associations and drug-disease relationships were used to develop a systematic drug repositioning method and to suggest, for instance, an antidiabetic effect for drugs causing porphyria.

On the other hand, other studies focus on understanding how side effects occur. As described above, relationships may exist between side effects and drug targets. Moreover, the link between chemical structure and side effects was shown by Scheiber et al. [7]. From a more mechanistic point of view, Lee et al. [8] showed that side effects can be correlated with the biological processes in which the drug targets are involved. For instance, they showed that nausea is correlated to an up-regulation of the deaminase activity. A very recent paper aims at predicting the side-effect profiles of molecules based on their chemical structures (defining the chemical space) and the information of their target proteins (defining the biological space) [9]. The so-called side-effect profile of a molecule is simply defined as its binary fingerprint with respect to the side-effect terms. However, such earlier studies have several limitations. For example, (i) they consider only individual side effects, and ignore the fact that often more than one side effect is associated with a drug, (ii) the biological space is over-simplified, and (iii) the resulting prediction models are “black boxes” which do not provide any explicit and reusable knowledge.

Here, we study in a systematic way drug side-effect associations, and we propose a method for identifying and characterizing side-effect profiles (SEPs) shared by several drugs.

**Figure 1:**
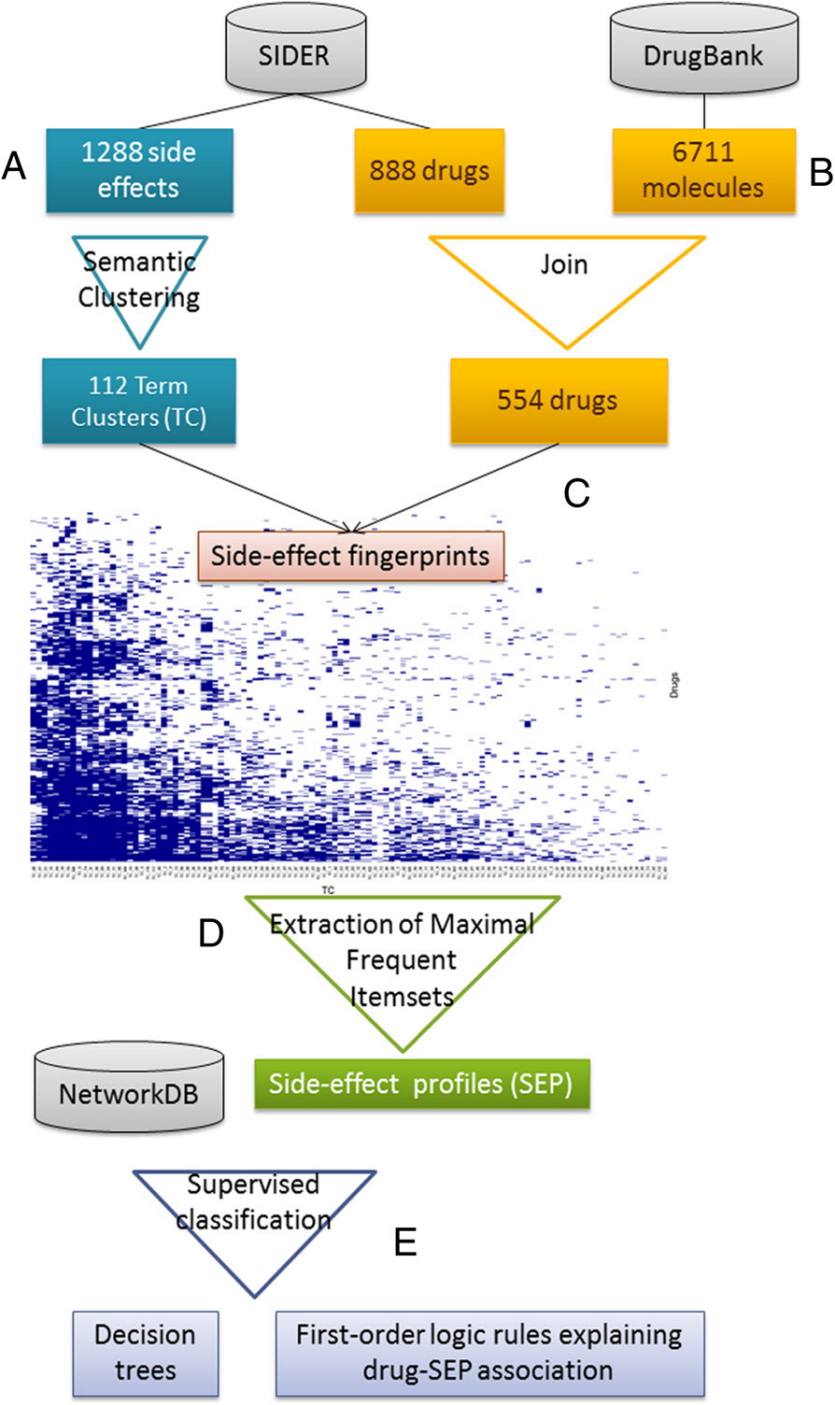
**Overview of our approach for characterizing drug-SEP associations.** Terms used for describing side effects in SIDER DB are grouped using a semantic similarity measure in order to build Term Clusters or TCs **(A)**. Drugs are mapped to DrugBank in order to retrieve information about drugs themselves and their targets **(B)**. TCs are associated to drugs to represent each drug by a side-effect fingerprint **(C)**. SEPs are extracted as maximal frequent itemsets from side effect fingerprints **(D)**. Two machine-learning methods are used to characterize each SEP in terms of drug and target properties **(E)**.

Our approach is composed of five main steps, as illustrated in Figure 1. The first step (Figure 1A) consists of grouping the terms used for side effects in SIDER using a semantic similarity measure in order to build Term Clusters (TC) corresponding to groups of semantically related SEs [10]. In parallel, drugs from SIDER are mapped to DrugBank in order to retrieve information about drugs themselves and their targets (Figure 1B). Then, TCs and drugs are associated in order to represent each drug by a side-effect fingerprint (Figure 1C). SEPs are extracted as maximal frequent itemsets from side effect fingerprints (Figure 1D). The aim is then to characterize each SEP in terms of drug and target properties. This can be addressed as a supervised classification task. Two machine-learning methods are chosen for this task: Decision Trees (DTs) and Inductive Logic Programming (ILP) (Figure 1E). These two methods provide easily readable results which can then be exploited for understanding SEPs. Decision trees use a single table as input in which each row corresponds to a drug and each column to a drug descriptor. Inductive Logic Programming uses relational descriptors to learn a first-order-logic concept definition from observations. Relational descriptors encoding characteristics of both drugs and their targets are retrieved from our “NetworkDB” integrated database, which is built from several data sources including DrugBank, UniProt, KEGG, and GO. The models obtained for a set of selected SEPs with these two machine-learning methods are then evaluated by cross-validation and tested directly with new drugs. Finally, some elements are provided for model interpretation.

## Methods

### The NetworkDB resource

**Figure 2:**
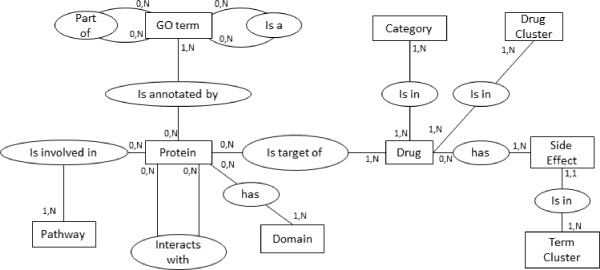
**NetworkDB conceptual model.** In this entity-relationship schema, entities are in boxes and relationships in ellipses.

NetworkDB is a relational database which integrates data about molecules and their targets. These data are collected from various public data sources mentioned in the following sections. Figure 2 shows the conceptual model of the database.

#### Chemical space: drugs and their properties

The SIDER database contains drug side-effect relationships [3]. DrugBank is used to collect data such as categories and targets [11]. The join between SIDER and DrugBank is based on the PubChem Compound identifier given by SIDER and DrugBank. A total of 554 drugs from SIDER are referenced in DrugBank v3.0.

Each drug is described by its category and a set of clusters it belongs to. In fact, various structural representations and associated similarity measures were used to cluster drugs. The first similarity measure is based on SMILES representation. The SMILES codes are converted thanks to Open Babel program into fingerprints which allows linear and ring substructures to be identified [12]. Then, the structural similarity between two molecular fingerprints is calculated using the Tanimoto measure. In addition, we calculated three other similarity scores using spherical harmonics representation of molecules. This parametric representation of macromolecular surface was originally proposed and applied by Ritchie and Kemp [13] and Cai et al. [14]. The proprietary program HPCC (Harmonic Pharma) supports three variants of the spherical harmonic representation. HPCCgeo uses spherical harmonic coefficients (shape information) to calculate similarity between drugs, HPCCchem is based on chemical properties mapped on the spherical harmonic representation, and HPCCcombo combines shape and chemical information. Ward’s method is used to perform four hierarchical clusterings of drugs [15]. The optimal numbers of clusters is determined by the method of Kelley and al. [16]. Thus, 60 clusters are obtained with Tanimoto, 53 with HPCCgeo, 21 with HPCCchem and 34 with HPCCcombo measures.

Drug categories are retrieved from DrugBank. These categories are mapped on the descendants of three MeSH concepts, namely “Molecular Mechanisms of Pharmacological Action” (*D27.505.519*), “Physiological Effects of Drugs” (*D27.505.696*) and “Therapeutic Uses” (*D27.505.954*).

#### Biological space: proteins and their properties

Drug targets are extracted from both DrugBank and PDB [17]. The outer join between PDB and DrugBank (retaining all DrugBank targets) is based on SMILES code identity. Drug targets are associated with their UniProt accession numbers. Thus, 768 targets are collected, representing an average of four targets per drug. Then, target annotations are retrieved from different databases. Protein-protein interactions are retrieved from the IntAct database [18] and 5959 interactions were collected which correspond to 2827 new proteins. For all the proteins (drug targets and their interactants), 1403 pathway names are extracted from the KEGG database and the Pathway Interaction Database which integrates data from NCI-Nature, BioCarta and Reactome [19,20]. For the same proteins, GO terms are also collected from QuickGO database [21]. Thus, 6494 GO terms annotating the 3595 proteins are stored in NetworkDB. Moreover, the “is_a” and “part_of” relationships between GO terms are stored in NetworkDB. Finally, 4650 protein domains associated with the targets and their interactants are retrieved from InterPro [22].

### Grouping side-effect terms into term clusters

Side effects are extracted from SIDER. As shown previously [23], the use of all terms describing side effects in SIDER (about 1500) impairs the execution of data mining programs and produces numerous and redundant patterns which are inappropriate for expert interpretation. As SIDER side effects terms belong to the Medical Dictionary for Regulatory Activities [24], a semantic similarity between these terms can be calculated based on the structure of MedDRA [10]. Next, a hierarchical clustering method is applied to obtain 112 Term Clusters (TCs) which are then validated by experts [23]. For instance, TC named 65_Dermatitis is the 65th TC and has Dermatitis as representative term.

### Datasets

#### Association of drugs with side effects

**Figure 3:**
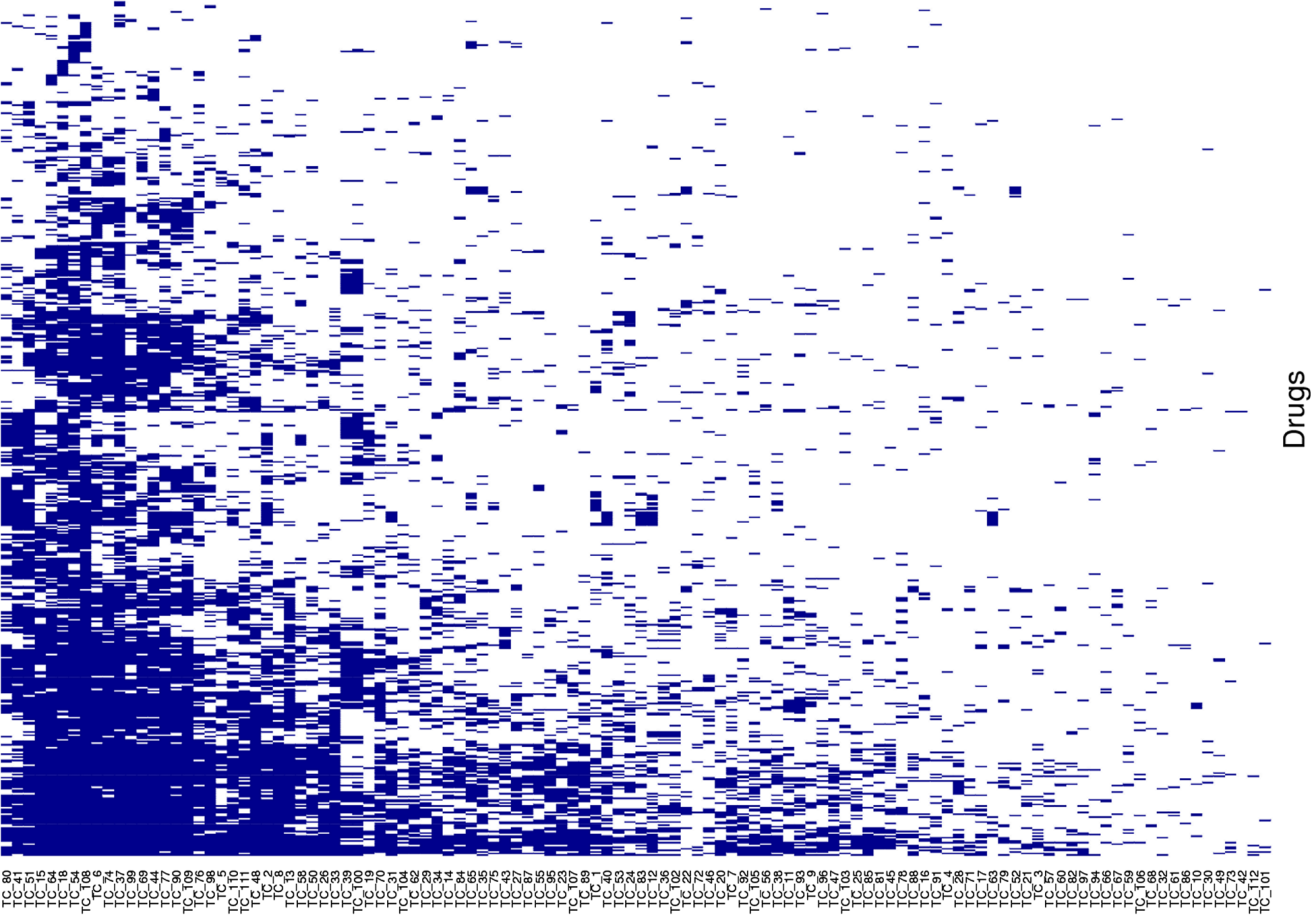
**Drug side-effect binary table.** This table is presented as a heatmap (produced with R) where rows and columns are grouped by distribution similarity. Each row represents the side-effect fingerprint of a drug and each column is a side-effect term cluster.

The association between drugs and TCs is an important step for the characterization of drugs sharing side effects. As the TC size varies from 2 to 59 terms, it seems consistent to use a heuristic procedure depending on the TC size. Let *k*_*i*_ be the number of terms in *TC*_*i*_ and *n*_*i*_ be the minimal number of side effects required for assigning *TC*_*i*_ to a drug. Considering *n*_*i*_ = 1 for any *TC*_*i*_ results in a very loose association yielding a very dense binary table hampering further computation, whereas considering *n*_*i*_ = *k*_*i*_ for any *TC*_*i*_ results in a very stringent association which might skip over important drug side effects. In fact a trade-off between these two extreme solutions is required. Grouping the *k*_*i*_ values into 5-range intervals with the last interval from 21 to 59 allows to set up a simple association procedure ranging *n*_*i*_ from 1 to 5. The resulting association between drugs and TCs is shown in Figure 3 where each row represents the side-effect binary fingerprint associated with a drug. This binary table (drug ×TC) is then used to discover interesting side-effect profiles defined here as the longest combinations of TCs shared by significant sets of drugs.

#### Single-table datasets

Single table datasets designed for DT learning represent each drug by an attribute-value vector. Four types of descriptors retrieved from NetworkDB are used to generate these attributes: the first is the class information, *i.e.* the studied SEP, the second one includes drug categories, the third one lists all drug targets with for each target, three attributes referring to the type of action of the drug (activation, inhibition and other) and the fourth concerns clusters of similar drugs according to the four similarity measures described above. Because of target and category multiplicity, the total dimension of this dataset varies between 741 and 924 depending on the SEP.

#### Relational datasets

Relational datasets designed for Inductive Logic Programming (ILP) consist in a set of tables extracted from NetworkDB describing drugs properties and background knowledge. Drugs properties are the same as in the single-table dataset, *i.e.* categories, targets and clusters. Background knowledge includes GO annotations, domain composition, interactants and pathways of each drug target. Relationships between GO terms constitute an additional table.

### Data mining

#### Maximal frequent itemsets

In a binary table (object ×attribute), a frequent itemset is a group of attributes shared by a number of objects greater than a threshold support. A frequent itemset is considered as a maximal frequent itemset (MFI) if all its proper supersets are not frequent [25]. It follows that two maximal frequent itemsets (MFIs) cannot be shared by a number of objects greater than the threshold support. In our case, MFIs are the largest combinations of TCs shared by a number of drugs greater than 100. This threshold was chosen as a trade-off between high values yielding short MFIs limited to one or two TCs and low values yielding numerous MFIs covering only a few molecules. MFIs are extracted from the binary table (Figure 2) using the Coron program [26] after excluding TCs which cover more than 50% of the molecules.

#### Decision trees

Decision tree (DT) construction is a machine-learning method which uses (object ×attribute) table to classify objects. Results given by this method are easily readable. Decision trees are built here with the J48 implementation of C4.5 tree learner in the Weka toolbox using single table datasets converted into the ARFF format [27]. We use the default parameters except for two of them: we use *minNumObj* = 5 and *binarySplits* = *true*.

#### Inductive Logic Programming (ILP)

ILP is a machine-learning method which uses relational data as input and has been successfully applied to various areas including bioinformatics [28-30]. It allows us to learn a concept definition from observations, i.e, a set of positive examples (E+) and a set of negative examples (E-), and background knowledge (B) [31]. The ILP experiments produce theories as sets of first-order logic rules. They where conducted here with the Aleph Program [32]. Many parameters can be tuned for theory construction. The three main parameters are the *min-pos*, the *noise* and the *induce-type*. The *min-pos* parameter is the minimal number of positive examples that a rule must cover. The *noise* corresponds to the maximal number of negative examples that an acceptable rule may cover (in our case, one is never sure that a drug does not have a given side effect). The third parameter is *induce-type* which directs theory construction. When this parameter is set to *induce-cover*, overlapping rules are produced (*i.e.*, a drug can be covered by several rules). Based on previous experience [33], we used the following settings: *min*-*pos* = 5, *noise* = 1 and *induce*-*type* = *induce*-*cover*.

### Model evaluation

#### Cross-validation

Both ILP theories and decision trees are evaluated with 10 runs of a 10-fold stratified cross-validation. DT cross-validation is performed with the Weka experimenter interface. For ILP, we took advantage of our recent integration of Aleph into the KNIME platform [34]. KNIME cross-validation meta-node is adapted for theory evaluation. An example is predicted as positive if it is covered by at least one rule. Each cross-validation assay yields a confusion matrix counting true and false positives, as well as true and false negatives. Each assay is then evaluated by the calculation of accuracy (ratio of correctly classified instances), specificity (true negative rate) and sensitivity (true positive rate).

#### Direct test

Theories and decision trees are also evaluated by direct test. Drugs used for testing are those present in SIDER 2 and DrugBank (v3.0) but not present in SIDER. For these drugs all descriptors are retrieved and stored in the NetworkDB. Furthermore, the reports of FAERS from 2004 to 2011 were imported as a database and used as an external information source for checking the false positives predicted by our models. We consider that a molecule is associated with a SEP in FAERS if for each TC of the SEP there is at least one report that states that the molecule is the primary suspect of an observed side effect belonging to the TC. Our checking procedure is just an anticipation as it relies on the fact that updating the package insert of a drug (stored in SIDER) requires that sufficient amount of adverse effect incidents occur (especially for new drugs).

## Results and discussion

### Overall distribution of side effects

A drug is associated with a TC (group of semantically related side effects) if it is annotated by a minimum number of side effects of this TC (see Methods). The resulting binary table is shown in Figure 3, where each row represents the side effect fingerprint of one of the 554 drugs considered here, and each column represents one of the 112 TC. In this representation, drugs and TCs have been grouped by distribution similarity. On the right part of the figure, we can see TCs associated with a limited number of drugs, whereas highly represented TCs are on the left. In the same way, drug fingerprints involving few TCs are on the top of Figure 3 and drugs with high number of TCs are on the lower part. Zooming on adjacent columns reveals that some TCs seem to be frequently associated with the same drugs as for example the pair TC 39_Stevens-Johnson_syndrome and TC 100_Erythema_multiforme.

However, apart from providing a general idea about the complexity of TC association with drugs, this visualization cannot be exploited easily. More precise information can be retrieved by querying NetworkDB. For example, the maximal number of TCs associated with a drug is 89 for the ropinirole (an anti-Parkinson agent). Conversely, 18 drugs are associated with only one TC. For instance, bretilium (an anti-hypertensive agent) is only associated with TC 110_Shock. From the TC point of view, the number of drugs associated with a TC ranges from 1 to 410. The 13 TCs covering more than 50% of the molecules are excluded in the rest of the study.

### Side-effect profiles

The overall intuition provided by Figure 3 is that groups of TCs shared by drugs exist and should be extracted. In fact, extracting patterns from such binary table is the purpose of itemset search algorithms [35]. We thus perform MFI extraction and we define side-effect profiles (SEPs) as maximal groups of TCs covering at least 20% of the drug set (110 drugs). The resulting 26 SEPs are listed in Table 1. Regarding length, 3 SEPs have only one TC, 13 combine 2 TCs, 9 combine 3 TCs, and only one combines 4 TCs. These 26 SEPs concern 372 molecules (67% of the drug set) and involve 18 distinct TCs of which the most frequent are 99_Headache and 90_Feeling_abnormal which appear 8 times each, whereas 7 TCs appear in only one SEP. These 26 most frequent SEPs are considered in the rest of the study. By construction, although two SEPs can have common TCs, they cannot cover more than 100 molecules in common.

**Table 1:** **Maximal frequent itemsets covering 20% of drugs (support) extracted from the drug*×*TC table**

**SEP**	**Profile composition**	**Support**	**Avg overlap**
SEP_1	41_Leukopenia, 90_Feeling_abnormal, 99_Headache	123	69
SEP_2	90_Feeling_abnormal, 99_Headache, 110_Shock	123	73
SEP_3	58_Gout	120	60
SEP_4	70_Pneumonia, 99_Headache	117	71
SEP_5	110_Shock, 111_Infection	117	68
SEP_6	76_Asthma, 90_Feeling_abnormal, 99_Headache	117	68
SEP_7	65_Dermatitis	116	53
SEP_8	2_Haemorrhage, 76_Asthma	115	65
SEP_9	41_Leukopenia, 76_Asthma	115	62
SEP_10	48_Rhinitis, 99_Headache, 111_Infection	115	69
SEP_11	41_Leukopenia, 110_Shock	114	66
SEP_12	39_Stevens-Johnson_syndrome, 41_Leukopenia, 100_Erythema_multiforme	114	52
SEP_13	41_Leukopenia, 48_Rhinitis	113	67
SEP_14	99_Headache, 100_Erythema_multiforme	113	56
SEP_15	31_Lymphadenopathy	112	59
SEP_16	70_Pneumonia, 90_Feeling_abnormal	112	71
SEP_17	41_Leukopenia, 70_Pneumonia	112	64
SEP_18	76_Asthma, 111_Infection	112	64
SEP_19	80_Jaundice, 100_Erythema_multiforme	112	45
SEP_20	41_Leukopenia, 111_Infection	111	63
SEP_21	8_Haematuria, 90_Feeling_abnormal, 99_Headache	111	68
SEP_22	13_Pyrexia, 33_Musculoskeletal_discomfort, 48_Rhinitis, 99_Headache	111	69
SEP_23	13_Pyrexia, 70_Pneumonia	110	69
SEP_24	48_Rhinitis, 90_Feeling_abnormal, 110_Shock	110	70
SEP_25	13_Pyrexia, 90_Feeling_abnormal, 110_Shock	110	70
SEP_26	48_Rhinitis, 90_Feeling_abnormal, 111_Infection	110	69

Avg overlap: average of overlap size between the SEP and other SEPs.

### Characterization of frequent SEPs

Our hypothesis is that a SEP shared by a large number of drugs can be explained in terms of drug properties and background knowledge. Thus, two machine-learning methods, decision trees and ILP, are applied on the drugs associated with each SEP. For both methods, the positive examples are taken to be all the drugs associated with a SEP, and those drugs that are not associated with any of the TCs composing the SEP are taken as negative examples. Negative examples represent 60% of the learning set.

For each profile, classification efficiency is evaluated using a 10 ×10 cross-validation by accuracy (Acc), specificity (Spec) and sensitivity (Sens). The results presented in Table 2 show that for both methods, generated models are good classifiers with an average accuracy of 67% for DTs and 65% for ILP. For 23/26 SEPs, accuracy is better for DTs than with ILP mostly reflecting the higher specificity values obtained with DTs. On the contrary, sensitivity values are always higher with ILP than with DTs with only one exception for SEP_17 where ILP sensitivity value is 0.1 lower than DTs sensitivity. Thus, ILP provides more sensitive theories whereas DTs provide more specific models. In fact, sensitivity is probably more important than specificity for drug development as it is for medical diagnostic. Indeed, low sensitivity means that some SEPs can be skipped over, although they are truly associated with the tested drug. Thus, ILP theories display attractive qualities for SEP prediction. Five SEPs (1, 3, 12, 15, and 19) are particularly well characterized with ILP since sensitivity values are greater than 60%. The amount and quality of available data may explain the observed differences of results between SEPs. It should be noted that comparison with other reported methods is uneasy due to the fact that we aim to characterize and predict SEPs rather than isolated side effects. In fact the closest study is the one of Yamanishi et al. [9] whose objective is to predict isolated side effects using multi-class statistical methods. Therefore these authors do not produce comparable accuracy values.

**Table 2:** Evaluation of learning results by 10 × 10 stratified cross-validation of DT and ILP programs

**SEP**	**DT**			**ILP**		
	**Acc**	**Spec**	**Sens**	**Acc**	**Spec**	**Sens**
SEP_1	0.65	0.86	0.39	0.61	0.63	0.6
SEP_2	0.69	0.88	0.4	0.63	0.69	0.54
SEP_3	0.71	0.88	0.47	0.71	0.77	0.63
SEP_4	0.66	0.89	0.32	0.62	0.7	0.51
SEP_5	0.68	0.88	0.38	0.64	0.7	0.54
SEP_6	0.68	0.87	0.39	0.61	0.69	0.49
SEP_7	0.65	0.86	0.32	0.6	0.67	0.49
SEP_8	0.7	0.87	0.44	0.67	0.73	0.57
SEP_9	0.69	0.84	0.46	0.69	0.75	0.59
SEP_10	0.7	0.89	0.4	0.65	0.76	0.47
SEP_11	0.7	0.88	0.44	0.7	0.82	0.45
SEP_12	0.71	0.88	0.45	0.7	0.76	0.61
SEP_13	0.67	0.88	0.35	0.66	0.74	0.54
SEP_14	0.69	0.89	0.39	0.63	0.71	0.51
SEP_15	0.71	0.9	0.43	0.69	0.76	0.6
SEP_16	0.69	0.89	0.39	0.66	0.72	0.57
SEP_17	0.74	0.89	0.52	0.65	0.74	0.51
SEP_18	0.65	0.87	0.34	0.61	0.69	0.5
SEP_19	0.74	0.91	0.47	0.72	0.77	0.64 0
SEP_20	0.71	0.89	0.44	0.64	0.73	0.51
SEP_21	0.72	0.9	0.46	0.64	0.72	0.54
SEP_22	0.65	0.88	0.32	0.61	0.69	0.48
SEP_23	0.71	0.89	0.43	0.63	0.7	0.51
SEP_24	0.68	0.87	0.4	0.62	0.71	0.5
SEP_25	0.71	0.9	0.43	0.65	0.72	0.56
SEP_26	0.69	0.88	0.4	0.62	0.69	0.52
Average	0.67	0.83	0.43	0.65	0.72	0.54

Acc: accuracy, Spec: specificity, Sens: sensitivity.

Table 3 shows the results obtained with the set of test molecules. Among the novel drugs present in SIDER 2, only 20 are associated with at least one of the 26 studied SEPs. These drugs have been tested with decision trees and ILP theories obtained for each SEP. The total number of drugs in the test set that are associated which each SEP is indicated (column Positives) and compared to the true positive values (TP columns) obtained with test set using either DT model or ILP theory relative to this SEP. Clearly the prediction results are better with ILP theories than with DTs. Indeed 22 true positives (covering 16 SEPs) were detected with ILP theories whereas only 9 true positives (covering 8 SEPs) were detected with DTs. The number of false positives are also reported for each SEP and each model (FP columns). The checking procedure was applied on false positives and the number of confirmed molecules according to FAERS is reported (FAERS columns). Thus, 33 molecules were extracted for ILP theories versus 37 for DTs raising the total number of probable true positives to 55 for ILP and 46 for DTs. Nevertheless, as the variability in cross-validation results suggest, many positive molecules still escape prediction especially for three SEPs: SEP_2, SEP_7, and SEP_21 with both DTs and ILP theories.

**Table 3:** Direct testing results with 20 new molecules

**SEP**	**Positives**	**DT**			**ILP**		
		**TP**	**FP**	**FAERS**	**TP**	**FP**	**FAERS**
SEP_1	4	0	5	1	2	3	1
SEP_2	11	1	2	1	0	1	1
SEP_3	2	0	3	1	0	5	1
SEP_4	3	0	3	1	1	2	1
SEP_5	5	0	2	1	1	2	1
SEP_6	5	1	5	3	2	3	1
SEP_7	15	2	2	1	2	1	1
SEP_8	4	1	1	1	1	3	1
SEP_9	3	0	3	2	0	3	1
SEP_10	5	0	1	0	0	3	1
SEP_11	4	1	3	2	1	3	1
SEP_12	0	0	5	1	0	4	1
SEP_13	4	0	6	2	1	4	1
SEP_14	4	1	0	0	1	5	2
SEP_15	1	0	2	1	0	5	2
SEP_16	3	0	6	2	2	7	3
SEP_17	1	0	5	3	0	2	1
SEP_18	2	0	3	1	0	2	1
SEP_19	1	0	4	2	0	5	2
SEP_20	3	0	3	1	1	4	1
SEP_21	8	1	2	1	1	2	1
SEP_22	5	0	3	1	1	5	1
SEP_23	3	0	3	1	1	4	1
SEP_24	5	1	4	2	2	5	2
SEP_25	8	0	3	2	2	5	2
SEP_26	4	0	6	3	0	3	1

Positives: number of positive examples in the test set according to SIDER, TP/FP: number of predicted true/false positives, FAERS: number of fished out molecules based on FAERS data.

### Interpretation of decision trees and theories

Quantitative characteristics of DT models and ILP theories for the 26 selected SEPs are presented in Table 4 (the decision trees and ILP theories are available at http://plateforme-mbi.loria.fr/side-effect-profiles).The first observation concerns model coverage. We can see that in average 83% of the drugs are covered by at least one rule in an ILP theory whereas DT models cover in average only 58% of the drugs composing the learning set. The second observation is the use of almost all descriptor types in each DT model or ILP theory. The most represented descriptors are drug categories and clusters for DTs, respectively drug targets and GO terms for ILP theories. This illustrates the importance of using background knowledge about drug targets and GO semantic relationships for the characterization of SEPs.

**Table 4:** Quantitative characteristics of DT models and ILP theories

	**DT (# nodes per model)**		**ILP (# rules per theory)**	
	**Avg (min-max)**	**% total**	**Avg (min-max)**	**% total**
Model coverage (%)	58 (32–67)	-	83 (77–88)	-
Model size	11 (6–15)	-	33 (16–40)	-
**Drug descriptors**				
Categories	4 (1–7)	34	6 (2–13)	19
Targets	3 (0–5)	26	30 (23–39)	90
Clusters	4 (1–9)	40	9 (4–14)	27
**Target descriptors**				
GO terms	NA	NA	24 (16–31)	73
Domains	NA	NA	1 (0–2)	1
Interactions	NA	NA	8 (2–16)	24
Pathways	NA	NA	4 (1–8)	12
**GO relationships**	NA	NA	6 (3–9)	19

Model coverage is the percentage of positive examples covered, averaged over the 26 DT models and 26 ILP theories. Avg: average. Model size corresponds to the average number of nodes in a DT model or of rules in a ILP theory. Occurrence of each type of descriptor is estimated by counting the number of nodes (rules respectively) involving them (NA: not applicable).

It is worth noting that some rules contained in theories were confirmed using peer-reviewed literature. For example, considering the SEP_7 (65_Dermatitis) theory, rule 11 says that a drug is associated with this SEP if its target interacts with a protein belonging to the KEGG pathway “Focal adhesion” and to the PID pathway “Signaling events mediated by focal adhesion kinase” (Table 5). By searching the list of genes implied in dermatitis [36] and confronting them to the 2 pathways, we extract 7 genes (*THBS1*, *COL1A2*, *COL3A1*, *COL4A1*, *COL5A*, *ITGB4* and *LAMA5*) dysregulated in dermatitis which belong to the KEGG pathway “Focal adhesion”. In the same way, two genes (*BDKRB2* and *PTGFR*) are known to be dysregulated in dermatitis and belong to the “Neuroactive ligand-receptor interaction” KEGG pathway mentioned in rule 14. Finally, if we consider rule 16 we could verify that the gene *ERBB3* belonging to the “Endocytosis” KEGG pathway is indeed down regulated in dermatitis.

**Table 5:** Theory obtained for 65_Dermatitis SEP (SEP_7)

**Rule #**	**Condition part of the rule**	**P**	**N**
3	drug_has_target(A,B,inhibitor), goterm(B,’cellular response to insulin stimulus’)	15	1
18	drug_has_target(A,B,inhibitor), goterm(B,C), go_relation(C,part_of,go:21543)	13	1
1	drug_has_target(A,B,activator), interact(B,C), goterm(C,’central nervous system development’)	12	1
30	drug_has_target(A,B,inhibitor), interact(B,C), pathway(C,’BCR signaling pathway’,pid), drug_cluster(A,’17_quinine’,hpcc)	12	0
24	drug_has_target(A,B,inhibitor), interact(B,C), goterm(C,’translation’), interact(C,D)	10	1
20	drug_has_target(A,B,inhibitor), interact(B,C), pathway(C,’BCR signaling pathway’,pid), pathway(C,’EPO signaling pathway’,pid)	9	1
25	drug_has_target(A,B,activator), goterm(B,’lipid binding’), goterm(B,’ligand-dependent nuclear receptor activity’)	9	1
35	drug_has_target(A,B,activator), interact(B,C), goterm(C,’identical protein binding’), goterm(C,’DNA binding’)	9	1
6	drug_has_target(A,B,inhibitor), goterm(B,’protein homodimerization activity’), drug_cluster(A,’16_gliclazide’,hpcc)	8	0
8	drug_has_target(A,B,activator), interact(B,C), interact(C,’Serine/threonine-protein phosphatase 2A 55 kDa regulatory subunit B beta isoform’)	8	1
15	drug_has_target(A,B,inhibitor), goterm(B,’response to ethanol’), goterm(B,’signal transduction’)	8	1
19	drug_has_target(A,B,inhibitor), goterm(B,C), go_relation(C,is_a,go:8227), drug_cluster(A,’16_Flavoxate’,hpcombo)	8	0
31	drug_has_target(A,B,inhibitor), interact(B,C), interact(C,’Dedicator of cytokinesis protein 1’)	8	0
5	drug_has_target(A,B,activator), goterm(B,’receptor activity’), interact(B,C), goterm(C,’mitosis’)	7	1
10	drug_has_target(A,B,inhibitor), goterm(B,C), go_relation(C,is_a,’cation channel activity’), goterm(B,’serotonin receptor activity’)	7	1
**14**	**drug_has_target(A,B,activator), pathway(B,’Neuroactive ligand-receptor interaction’,kegg), goterm(B,’transcription, DNA-dependent’), goterm(B,’signal transduction’)**	**7**	**0**
**16**	**drug_has_target(A,B,inhibitor), pathway(B,’Endocytosis’,kegg)**	**7**	**0**
21	drug_has_target(A,B,activator), interact(B,C), interact(C,’RNA polymerase-associated protein CTR9 homolog’)	7	1
22	drug_has_target(A,B,inhibitor), pathway(B,’Role of Calcineurin-dependent NFAT signaling in lymphocytes’,pid), goterm(B,’signal transduction’)	7	1
23	drug_has_target(A,B,inhibitor), interact(B,C), domain(C,’ Protein synthesis factor, GTP-binding’)	7 1	
28	drug_cluster(A,’7_marinol’,hpcombo)	7	1
7	category(A,’Topoisomerase Inhibitors’), drug_has_target(A,B,inhibitor), goterm(B,’transferase activity’)	6	1
12	drug_cluster(A,’29_norfloxacin’,hpcf)	6	1
17	category(A,’Cyclooxygenase 2 Inhibitors’), drug_cluster(A,’2_estazolam’,hpcc)	6	0
32	drug_has_target(A,B,activator), goterm(B,’inflammatory response’), goterm(B,’protein binding’)	6	0
2	category(A,’Serotonin Uptake Inhibitors’)	5	0
4	drug_has_target(A,B,inhibitor), goterm(B,’synapse assembly’), drug_cluster(A,’14_fentanyl’,hpcombo)	5	1
9	drug_has_target(A,B,activator), goterm(B,’protein heterodimerization activity’), goterm(B,’cell-cell signaling’)	5	1
**11**	**drug_has_target(A,B,other), interact(B,C), pathway(C,’Focal adhesion’,kegg), pathway(C,’Signaling events mediated by focal adhesion kinase’,pid)**	**5**	**0**
13	category(A,’HIV Protease Inhibitors’), drug_has_target(A,B,inhibitor), goterm(B,C), go_relation(C,is_a,D), go_relation(D,is_a,’catalytic activity’)	5	1
26	drug_has_target(A,B,inhibitor), goterm(B,’heart development’)	5	1
27	drug_has_target(A,B,inhibitor), goterm(B,C), go_relation(C,is_a,go:65008), drug_cluster(A,’55_thiothixene’,tanimoto)	5	0
29	category(A,’HIV Protease Inhibitors’), drug_has_target(A,B,inhibitor), goterm(B,’oxidation reduction’)	5	0
33	drug_has_target(A,B,other), goterm(B,C), go_relation(C,is_a,go:51240)	5	1
34	drug_has_target(A,B,inhibitor), goterm(B,C), go_relation(C,is_a,’binding’), drug_cluster(A,’27_quinine’,hpcombo)	5	1

The condition parts of the 35 rules contained in SEP_7 theory are given with the number of positive (P) and negative (N) covered examples. The 3 rules confirmed using peer-reviewed literature are in bld. Rules are ordered by number of positive covered examples. The 8 predicates are defined as follows: Drug_has_target(A, B, inhibitor/activator) : drug A activates/inhibits protein B; goterm(B, G): protein B is annotated by GO term G; go_relation (G1, R, G2): the relationship between GO terms G1 and G2 is R; interact(B,C): protein B interacts with protein; pathway(B, P): protein B is involved in pathway P; drug_cluster(A,K,M): drug A is member of cluster K obtained using method M; category(A,T): drug A belongs to category T; domain(B,D): protein B is composed of domain D.

Finally, from a more global point of view the drugs can be represented according to the rules they satisfy resulting in a drug ×rule binary table. This table constitutes a kind of abstraction of the initial drug ×TC binary table (Figure 3) based on extracted knowledge. Interestingly this new representation leads to improved clustering results for the drug set (not shown) and could be further exploited for prediction studies of particular SEPs.

## Conclusions

Our study proposes an integrative machine-learning approach for predicting side-effect profiles (SEPs) and understanding their mechanisms. We integrate drug characteristics and background knowledge such as functional annotation, interactions and pathways in a relational database. An extensive learning set is built by associating drugs with clusters of side effects (TCs) according to SIDER information. Our first contribution consists of extracting SEPs from this complex table of fingerprints as the longest groups of TC shared by more than one hundred drugs. We also set up two machine-learning methods, namely decision trees and inductive logic programming in order to learn which combination of properties of drugs and their targets leads to a given SEP. After evaluating the learning models, our general observation is that ILP models have a higher sensitivity than DT models. Because higher sensitivity means predicting fewer false negatives, this means that ILP predicts SEPs more often than decision trees. This was confirmed on a small test set including a checking procedure using FAERS as external and complementary information source. Indeed, more sophisticated prediction procedures can be designed integrating FAERS and based on selected rules. This should improve the prediction accuracy at least for specific SEPs displaying good quality data. The results obtained with ILP also show that background knowledge is well exploited during rule induction. Thus, in addition to targets, chemical structure and biological process annotation already studied by other groups [4,7,8], we show that information about pathways, protein-protein interaction and to a lower extent protein domains also plays an important role in side effect characterization. Further experiments may include other types of background knowledge such as clinical data and/or polymorphisms.

In our approach we characterize SEPs instead of individual TCs. Indeed as drugs are frequently associated with more than one TC, studying separately each TC implicitly assumes that side effects occur independently one from the other. This likely corresponds to a simplified view of side-effect occurrence and the existence of SEPs shared by more than 20% of the drug set strongly suggests that side effects are correlated. Moreover our approach can be applied to any user-defined SEP or TC of interest.

We believe that our approach represents a valuable methodology for understanding and predicting side-effect profiles. Our results suggest that the first-order logic theories can already be used during the drug discovery process in order to early anticipate side-effect apparition and thus decrease the attrition rate.

## Availability of supporting data

All decision trees and ILP theories are available at http://plateforme-mbi.loria.fr/side-effect-profiles.

## Abbreviations

Acc: Acccuracy; DT: Decision tree; ILP: Inductive logic programming; MFI: Maximal frequent itemset; Sens: Sensibility; SE: Side effect; SEP: Side effect profile; Spec: Specificity.

## Competing interests

The authors declare that they have no competing interests.

## Authors’ contributions

EB participated to the conception and design of the study and acquisition of data. He carried out the machine learning experiments. RG designed and developed programs for automatizing machine learning experiments and cross validations. GM carried out the clustering experiments on molecules. ASK and MS participated in the conception of the study and the interpretation and critical analysis of the results. MDD and MST conceived the study and carried out its design and coordination and helped EB to draft the manuscript. All authors read and approved the final manuscript.
